# A comparative study of energy and carbon efficiency for emerging countries using panel stochastic frontier analysis

**DOI:** 10.1038/s41598-019-43178-7

**Published:** 2019-04-30

**Authors:** Taeyoung Jin, Jinsoo Kim

**Affiliations:** Hanyang University, Department of Earth Resources and Environmental Engineering, Seoul, 04763 Republic of Korea

**Keywords:** Environmental economics, Energy efficiency

## Abstract

The demand for energy has been increasing significantly worldwide. Consequently, carbon emissions have accelerated, since energy usage involves carbon dioxide. Given that the available energy has quantitative restriction feature, efficient usage of energy becomes crucial. Energy efficiency is expected to improve over time with technological advancements. However, the adoption of low-carbon energy technology caused by the growing concern about carbon emissions may actually offset energy efficiency, owing to the higher cost compared with traditional energy methods. We conducted a stochastic frontier analysis to examine energy efficiency in the views of both economic and ecological aspect view during 1995–2016 for 21 emerging countries selected from Morgan Stanley Capital International, using energy consumption, economic complexity index and the other factors of production based on the Cobb-Douglas production function. Mexico was identified as one of the most energy-efficient countries; however, Mexico could not be classified as real energy efficient, as it demonstrated the highest carbon inefficiency level. We also categorized countries demonstrating increased economic energy efficiency and decreased carbon inefficiency as frontier country, and identified six such countries.

## Introduction

The demand for energy has been rising rapidly worldwide. According to the IEA^1^, this demand will continuously increase until 2040, despite the promotion of energy consumption reduction policies for environment. Switching to clean energy technology is being encouraged to reduce carbon emissions, which is emerging as a serious issue. Although it is well-recognized that technological advancement improves energy efficiency by decreasing costs and increasing productivity, the recent energy technology development regime, such as the Clean Development Mechanism (CDM), and high R&D expenditure for renewable energy might hinder energy efficiency improvement in terms of economic aspect^2^. Economic energy efficiency is written as energy efficiency hereafter. Lund^3^ also agree with that the improvement of efficiency must precede expansion of renewable energy in energy mix. Furthermore, Menegaki^4^ revealed the evidence that renewable energy pioneer countries are not most technical efficient economies. Menegaki^4^ adopted DEA to estimate the technical efficiency of European countries. The DEA efficiency score results show that countries using renewable energy remarkably tend to have medium to low efficiency.

Although we are sure that this is caused by the hidden cost from traditional energy^5,6^, renewable energy costs must be comparable level of traditional energy^7^. Thus, accurate energy efficiency estimation is essential to provide useful information to policymakers.

Traditional energy efficiency methods usually consider energy intensity as a proxy for energy efficiency, which implies that the economic output represents productivity. However, it is not appropriate to measure energy efficiency using energy intensity, since the latter does not consider the economic structure but only total energy consumption and economic output^8,9^. Thus, this measure has proven to be controversial. Changes in energy intensity imply not only changes in energy efficiency, but also changes in economic structure. For instance, a country characterized by a capital-intensive economy tends to have high energy intensity. To overcome this limitation, we estimate energy efficiency using the Stochastic Frontier Analysis (SFA), which can reflect various components of economic output.

However, even if we estimate energy efficiency using SFA, we would face a significant problem. Some countries may achieve cost reduction by using relatively inferior good such as coal, which may represent high energy efficiency. In this scenario, the following question arises: Does this kind of efficiency imply true efficiency? It is clear that clean energy sources, which are represented by renewable energy, are more expensive than traditional fossil fuels. Thus, we must consider the ecological aspect when estimating energy efficiency using economic output generated by an energy input^10^. In addition, the literatures covering emerging countries are relatively few. Emerging countries are a kind of developing countries, which have per capita income lower than average and high volatility. These markets should be reviewed through empirical analysis since they have high change potential. Depending on the understanding current situation and relevant policy making, the global energy transition and climate change will differ.

Global warming has been a serious concern and many international organizations are working toward resolving this issue. For example, the Intergovernmental Panel on Climate Change (IPCC) has been publishing annual reports and the United Nations Framework Convention on Climate Change (UNFCCC) has been holding annual conferences to assess the impact of global warming and options for its mitigation. Even though Paris agreement was successful, there still exists the concerns such as the temperature target^11^. Given the growing concern about carbon emissions, we decided to include carbon emissions in our analysis. We assumed that carbon can also be an output of energy input in this analysis, even though it is undesirable. Thus, it can be estimated using SFA. In this analysis framework, a country using relatively cheap but high carbon-generating energy source may have both high energy efficiency and high carbon inefficiency scores.

The methodology adopted in this research is a panel version of SFA. Panel analysis allows the model to control for unobserved heterogeneity and estimate time-varying efficiency. The ordinary SFA is applied to cross-sectional data with a strong assumption of time-invariant technical efficiency. Furthermore, data without outliers have informative power when they are utilized with proper statistic tools. Accordingly, panel data analysis has more explanatory power than cross-sectional or time series data, since panel has one additional data dimension.

The purpose of this study is to estimate the energy efficiency and carbon emission inefficiency for 21 emerging countries. We chose emerging countries as the research subject for energy efficiency, since most of the energy demand growth is caused by emerging economies^12^. To the best of our knowledge, there is no research that examines energy efficiency in both views of economic and ecological aspect using the SFA method, particularly for emerging countries. In our analysis framework, the Cobb-Douglas production function is adopted to estimate energy and carbon efficiency. Economic output and carbon emissions are handled as output and unfavorable output in energy efficiency and carbon efficiency model, respectively.

The main contributions of this research are as follows. First, our study is novel in that we apply panel SFA, which allows to estimate time-varying efficiency levels, to country-wide analyses of emerging countries. Second, we considered energy and carbon efficiency together, reflecting the importance of climate change mitigation. Based on our results, we categorized subject countries and suggested policy implications accordingly. Finally, to overcome the limitations of traditional energy efficiency estimation using energy consumption and economic output, and to reflect the industrial structure of each country, we incorporated the economic complexity index (ECI) proposed by Hidalgo and Hausmann^13^ into our model.

The remainder of this paper is organized as follows. Section 2 introduces the existing literature on energy efficiency and carbon inefficiency. Section 3 presents the estimation procedure and the methodology. Section 4 discusses the data and empirical results. Finally, Section 5 concludes the study.

## Literature Review

There are two representative ways to estimate energy efficiency: Data Envelopment Analysis (DEA) and SFA. These can be categorized into non-parametric and parametric, respectively. Although there also exists semi-parametric methods, this is not well-used in energy efficiency estimation. SFA has more statistical power than DEA because this is a parametric method. Furthermore, SFA has no assumption on the model but just statistical part, unlike DEA has an assumption of linear projection. We decided to adopt SFA in our efficiency estimation and focused on the literatures using SFA. Numerous studies in the literature have analyzed energy efficiency with SFA. The scope of analysis varies from industry level to country level. A major part of the literature focuses on China^14–18^, as China’s energy efficiency has attracted significant interest among researchers.

Besides, the cases of Japan and Sweden have also been discussed in energy and carbon efficiency research^19–23^, given their economic firmness and high ecological efforts. In particular, Sweden was less affected by the European economic crisis compared with many other European countries, and the country has been primarily focusing on taxation of energy and carbon emissions in terms of energy efficiency policies^24^. Regarding Japan, they have their own energy efficiency target, which has been accomplished by the “setsuden” energy conservation project since 2011^25^.

Energy efficiency research for the United States also exists. Filippini and Hunt^26^ analyzed the residential sector’s energy efficiency for a panel of 48 states using stochastic energy demand frontier analysis. They considered various components, including energy consumption, income, and energy price by utilizing the functional form of demand. They also selected population, household size, and climate condition variables (Heating degree days and Cooling degree days). It is useful to consider various elements when we attempt to estimate energy efficiency since energy consumption and economic factors are determined by the interaction of diverse aspects.

Some studies cover a panel of several countries, rather than focusing on a single^19,27–29^. Filippini and Hunt^27^, Zhou *et al*.^29^, and Hu and Honma^19^ employed OECD countries as the subject of analysis. On the other hand, Marin and Palma^28^ analyzed energy efficiency of EU countries. Given that existing literatures chose such countries, we can conclude that previous studies on energy efficiency analysis mainly focused on developed countries. The selected variables in the existing literatures are diverse. The literatures using demand function utilized energy price variable. Filippini and Hunt^27^ used the most kinds of variables. They adopted energy price, economic growth, population, area size, climate factor, and value added by major sector such as industrial and service sector. The other literatures also utilized these factors or less than these. However, the endogeneity problem can be occurred in the econometric analysis when the high relationship between explanatory variables exists. Of course, more data without outliers can be more informative power but more variables indicate that the possibility to occur endogeneity problem will rise. Thus, we adopted only major variables which do not disturb each other.

Herrala and Goel^30^ studied global carbon dioxide efficiency using a stochastic cost frontier analysis of 170 countries. However, this study is rather different from the intention of ours. While we view carbon efficiency considering energy and the other factors of production, they constructed cost function consisting of GDP and population and estimated carbon dioxide frontier. Through that, Herrala and Goel^30^ estimated carbon efficiency. However, we insist that energy consumption be included when estimating carbon emissions. Relatively few studies about carbon efficiency have been published, compared with those covering energy efficiency. We should concentrate more on carbon emissions, since carbon emissions have increased alongside energy demand growth. Thus, we attempted to analyze energy efficiency and carbon emission and utilize those results to suggest policy implications comprehensively. For our analysis, we selected countries that have recently experienced a boost in economic growth and that have large emissions potential (emerging countries). These countries will be a interesting subject since they are still undergoing transition.

## Model Specification and Methodology

Our efficiency estimation model based on Cobb-Douglas production function. With the traditional factors of production, capital and labor, energy consumption and ECI are adopted as additional explanatory variables, as we mentioned in Section 1. Energy consumption is the key variable in our models. The Gross Domestic Product (GDP) and carbon emissions are utilized as dependent variable for the energy efficiency model and carbon inefficiency model, respectively. Each estimation model can be presented as follows:

<Model I – Energy efficiency model>

1 $$Y=f(E,K,L,ECI)$$

<Model II – Carbon inefficiency model>

2 $$CE=f(E,K,L,ECI)$$

where $E,\,K,\,L$ denote energy consumption, gross fixed capital formation, and labor force, respectively. $Y$ in the Model I represents the economic output (GDP) and $CE$ in the Model II indicates carbon emissions.

The SFA model can be classified into two categories: input-oriented and output-oriented^31^. The input-oriented SFA measures how much input exceeds comparing to the input to produce frontier output. On the other hand, the output-oriented SFA estimates how much output shortfall from the frontier^32^. In our analysis, both Model I and II use output-oriented model. For the model II, the farther from frontier line, the results show that the more efficient in the view of ecology since carbon emissions are undesirable goods.

Data Envelopment Analysis (DEA) is also widely used to estimate technical efficiency. However, since DEA is non-parametric methodology, SFA has statistical power than DEA by considering the measurement error and statistical random walk noise. Furthermore, DEA ignores the change along with time since DEA is applied to cross-section data. Thus, the panel version of SFA method is more useful than DEA.

The SFA methodology adopted in this research is the True Fixed Effect (TFE) model suggested by Greene^33^. We assume the individual characteristics of each country since our research subject, emerging countries, consists of heterogeneous group of countries. Individual characteristics can be reflected only when using fixed effect model. This methodology estimates the frontier and efficiency as follows:

3 $${y}_{it}={\alpha }_{i}+{x}_{it}\beta +{e}_{it}$$

where $i$ and $t$ denote cross-section and time-series dimension, respectively. ${e}_{it}$ represents the error term. The error term can be divided into two components: idiosyncratic error (${\upsilon }_{it}$) and technical inefficiency (${u}_{it}$). We assume that the technical inefficiency term follows half-normal distribution.

4 $$\begin{array}{c}{e}_{it}={\upsilon }_{it}-{u}_{it}\\ {\upsilon }_{it} \sim N(0,{\sigma }_{\upsilon }^{2})\\ {u}_{it} \sim N(0,{\sigma }_{u}^{2,+})\end{array}$$

The technical efficiency can be estimated through SFA following the framework proposed by Jondrow *et al*.^34^. This efficiency is equal to the ratio of the predicted dependent variable when technical inefficiency is zero (frontier, ${\widehat{y}}_{it}^{f}$) and the observed dependent variable (${y}_{it}$).

5 $$efficienc{y}_{it}=\frac{{\widehat{y}}_{it}^{f}}{{y}_{it}}=\exp (-\,{u}_{it}).$$

We conducted SFA and estimated energy efficiency and carbon inefficiency through the above procedure. The final estimation model can be expressed as follows:

6 $${Y}_{it}={\beta }_{1i}+{\beta }_{1E}{E}_{it}+{\beta }_{1K}{K}_{it}+{\beta }_{1L}{L}_{it}+{\beta }_{1ECI}EC{I}_{it}+{\upsilon }_{1it}-{u}_{1it}$$

7 $$C{E}_{it}={\beta }_{2i}+{\beta }_{2E}{E}_{it}+{\beta }_{2K}{K}_{it}+{\beta }_{2L}{L^{\prime\prime} }_{it}+{\beta }_{2ECI}EC{I}_{it}+{\upsilon }_{2it}\,-{u}_{2it},$$

where each variables are natural log transformed since Cobb-Douglas production function can be linear form by natural logarithm. In these equations above the variables are not a form of per capita. We compare the entire output against entire input for a country. Energy and carbon efficiency must be with gross data. Besides, the estimated coefficients with per capita variables are not statistically significant to explain the models. In short, Eqs (6 and 7) are estimated, and Jondrow *et al*.^34^’s technical efficiency estimation procedure is applied to measure the energy and carbon efficiency. As we mentioned above, the higher the efficiency score estimated by Eq. (7), the higher the carbon inefficiency. Thus, we consider efficiency results from Eq. (7) as inefficiency score. The empirical results from this analysis are shown in the next section.

## Data and Empirical Results

We collected macro-level data to investigate energy and carbon efficiency. Panel data for energy consumption in kilo tonnes of oil equivalent (ktoe) is collected from the energy balance of IEA^35^. Data on carbon emissions in kilo tonnes and economic complexity index are derived from the European Commission^36^ and the CID^37^, respectively. Gross fixed capital formation and GDP in constant 2010 US$ as a proxy of capital and economic growth, respectively, and labor force are derived from World Development Indicators^38^. ECI is the dimensionless variable suggested by Haumann and Hidalgo^39^. ECI is measured by the equation that how much a country exports the product to other countries with examining diversity and ubiquity. We used a balanced panel dataset for 21 emerging countries composed annually from 1995 to 2016.

The emerging countries were selected from Morgan Stanley Capital International (MSCI). MSCI have announced 24 emerging markets. MSCI’s market classification categorizes Brazil, Chile, China, Colombia, Czech Republic, Egypt, Greece, Hungary, India, Indonesia, Korea, Malaysia, Mexico, Pakistan, Peru, the Philippines, Poland, Qatar, Russia, South Africa, Taiwan, Thailand, Turkey, and United Arab Emirates (UAE) as emerging markets. Taiwan, Qatar, and UAE were excluded due to data unavailability. No ECI data are available for Taiwan. There are no capital and labor force data for Qatar and UAE. However, data for other variables are available. According to MSCI, 24 emerging markets have been selected by following conditions: (1) per capita income lower than average. (2) rapid growth, (3) high volatility, (4) less mature investment capital than the developed countries, (5) higher than average return for investors. The second and fifth characteristics are interconnected. Table 1 presents the descriptive statistics. Cross-section and time-series length are 21 and 22, respectively. The total observations are 462. These observations are enough to conduct TFE without losing explanation power of the model.

**Table 1:** Descriptive statistics.

Variable	Min	Max	Mean	Standard deviation	Units
Energy consumption (*E*)	10,938.00	2,991,431.00	227,122.70	445,417.28	ktoe
Capital (*K*)	12,484,892,787,88	4,414,250,000,000.00	205,344,342,288.19	484,322,236,041.21	constant 2010 US$
Labor force (*L*)	4,011,271.00	793,307,655.00	84,262,739.38	175,431,744.42	—
Economic complexity index (*ECI*)	−1.04	1.91	0.35	0.63	dimensionless
Economic growth (*Y*)	75,500,268,871.94	9,505,156,930,655.12	720,233,600,901.86	1,136,634,137,589.83	constant 2010 US$
Carbon emissions (*CE*)	25,629.73	10,546,277.00	649,981.16	1,535,556.04	kton

The empirical results for the Eqs (6 and 7) are shown in Table 2. The results show that the estimated coefficients are statistically significant, and the sign of each coefficient is intuitive.

**Table 2:** Estimated coefficients of the TFE models.

Explanatory variable	Model I	Model II
Energy consumption ($E$)	−3.150 (0.000)***	2.159 (0.037)***
Capital ($K$)	3.450 (0.000)***	0.042 (0.046)
Labor force ($L$)	0.516 (0.239)**	−0.315 (0.080)***
Economic complexity index ($ECI$)	−0.157 (0.311)	−0.677 (0.078)***
**Idiosyncratic error and technical inefficiency term**		
${\sigma }_{\upsilon }$	0.309 (0.113)***	0.241 (0.022)***
${\sigma }_{u}$	0.638 (0.051)***	0.053 (0.010)***
Lamda ($\lambda$)	0.484 (0.148)*	4.572 (0.028)***

Note: ***, **, and * denote rejection of the null hypothesis at the 1%, 5%, and 10% significance level, respectively.

The estimated coefficient corresponding to each variable can be interpreted as an elasticity coefficient, since all variables are transformed into natural logarithms. Most of estimated coefficients are statistically significant at the 1% significance level. The ECI coefficient in the Model I and capital coefficient of Model II are statistically insignificant.

For the energy efficiency model, a 1% increase in total energy consumption leads to 3.150% decrease of economic growth. The other variables, capital and labor force have a positive relationship with economic growth. A 1% increase in capital and labor input increase economic growth by 3.450% and 0.516%, respectively. In case of the carbon inefficiency model, energy consumption is shown to accelerate carbon emissions. If energy consumption increases 1%, carbon emissions grow to 2.159%. While capital has no significant impact on carbon emissions, labor force is decreasing 0.315% of additional emissions by 1% increase. Economic complexity also partially contributes carbon mitigation with 0.677% elasticity.

Tables 3 and 4 show that the average energy efficiency and carbon inefficiency scores over the period 1995–2016 from the estimation results of Eqs (6 and 7), respectively.

**Table 3:** Average energy efficiency score.

Country	Energy efficiency	Rank	Country	Energy efficiency	Rank
Brazil	0.762	1	Malaysia	0.726	18
Chile	0.736	15	Mexico	0.760	3
China	0.712	19	Pakistan	0.760	5
Colombia	0.727	17	Peru	0.744	10
Czech Republic	0.760	4	The Philippines	0.751	8
Egypt	0.760	2	Poland	0.734	16
Greece	0.739	13	Russia	0.706	21
Hungary	0.755	7	South Africa	0.742	11
India	0.738	14	Thailand	0.712	20
Indonesia	0.747	9	Turkey	0.742	12
Korea	0.759	6

**Table 4:** Average carbon inefficiency score.

Country	Carbon inefficiency	Rank	Country	Carbon inefficiency	Rank
Brazil	0.718	3	Malaysia	0.823	12
Chile	0.727	4	Mexico	0.893	21
China	0.732	5	Pakistan	0.852	15
Colombia	0.777	8	Peru	0.712	2
Czech Republic	0.881	19	The Philippines	0.804	10
Egypt	0.709	1	Poland	0.886	20
Greece	0.867	18	Russia	0.787	9
Hungary	0.861	16	South Africa	0.830	13
India	0.762	7	Thailand	0.755	6
Indonesia	0.835	14	Turkey	0.918	11
Korea	0.863	17			

Note: We ranked carbon inefficiency score in ascending order since a high inefficiency score indicates large amount of carbon emissions (unfavorable goods) at the same condition.

According to our empirical results, the country that has the highest energy efficiency score is Brazil followed by Egypt, Mexico, and Czech Republic. The country with the lowest energy efficiency score is Russia. Regarding carbon inefficiency, Egypt records the lowest carbon inefficiency score, which implies Egypt emits the smallest amount of carbon at the same energy usage and economic complexity conditions. However, these results are just average score for the period of 1995–2016. In 2016, the rank of energy efficiency and carbon inefficiency is rather different. On the other hand, Mexico has the highest carbon inefficiency score. This result provides useful implications given that Turkey is one of the most energy-efficient countries among the emerging countries, a high energy-efficiency score may not necessarily indicate true efficiency.

The average value, however, cannot be the representative, although it provides useful information. Thus, we investigated the difference in energy efficiency and carbon inefficiency growth between 1995 and 2016. This can explain whether the energy efficiency and carbon inefficiency increased or not through the sample period. Table 5 presents the results

**Table 5:** Energy efficiency and carbon inefficiency growth from 1995 to 2016.

Country	Energy efficiency growth	Carbon inefficiency growth	Country	Energy efficiency growth	Carbon inefficiency growth
Brazil	0.082	−0.335	Malaysia	0.414	−0.166
Chile	−0.142	−0.362	Mexico	−0.149	−0.015
China	−0.456	−0.548	Pakistan	0.097	−0.306
Colombia	−0.146	−0.173	Peru	−0.043	−0.528
Czech Republic	−0.119	−0.010	The Philippines	−0.292	−0.014
Egypt	−0.041	−0.555	Poland	−0.239	0.083
Greece	0.058	−0.042	Russia	−0.165	0.092
Hungary	−0.111	0.083	South Africa	−0.198	−0.109
India	−0.211	−0.378	Thailand	0.652	−0.500
Indonesia	−0.066	−0.230	Turkey	−0.193	−0.530
Korea	0.167	−0.293			

As shown in Table 5, 15 out of 21 countries demonstrated a decrease in energy efficiency over the entire sample period—Chile, China, Colombia, Czech Republic, Egypt, Hungary, India, Indonesia, Mexico, Peru, the Philippines, Poland, Russia, South Africa, and Turkey. Of the 15 countries, three have demonstrated an increase in carbon inefficiency—Hungary, Poland, and Russia—, which may be a problem. Energy efficiency is commonly believed to decrease when one country attempts to apply the clean energy system that produces low-carbon emissions. However, Hungary, Poland, and Russia demonstrate a decrease in energy efficiency and increase in carbon inefficiency simultaneously. This inverse-frontier movement of Hungary, Poland, and Russia can be explained by high economic complexity index. Their rankings among emerging countries are almost at the top. This means that when measuring energy and carbon efficiency, high economic complexity may adversely affect efficiency score. There is no country that demonstrates an increase in both energy efficiency and carbon inefficiency. This implies that rise of interests in climate change protect a country’s energy mix from adopting cheap energy sources.

We classify countries that demonstrate an increase in energy efficiency and decrease in carbon inefficiency as frontier countries, since they were able to accomplish the twin objectives. Our results suggest that Brazil, Greece, Korea, Malaysia, Pakistan, and Thailand come under this category.

As we discussed in the introduction section, energy intensity cannot fully explain the energy efficiency level of each country due to certain limitations. To reinforce this claim, we compare our efficiency score and energy intensity ranking. Table 6 presents a comparative analysis of energy efficiency ranked by SFA and energy intensity.

**Table 6:** A comparative analysis of energy efficiency ranking by SFA and energy intensity.

Country	SFA	Energy intensity	Country	SFA	Energy intensity
Brazil	1	2	Malaysia	18	14
Chile	15	6	Mexico	3	7
China	19	18	Pakistan	5	20
Colombia	17	3	Peru	10	4
Czech Republic	4	10	The Philippines	8	12
Egypt	2	16	Poland	16	11
Greece	13	1	Russia	21	21
Hungary	7	8	South Africa	11	17
India	14	19	Thailand	20	15
Indonesia	9	13	Turkey	12	5
Korea	6	9			

Some differences can be identified between energy efficiency rankings estimated with SFA and energy intensity. The major difference was detected in Russia and Brazil. The Energy efficiency ranking by SFA for both are 21st and 1st, respectively; however, they are ranked 2nd and 21st, respectively, by energy intensity. On the other hand, Colombia and the Philippines seem to have an opposite trend. This difference may be attributed to consider the other factors of production and economic structure considerations.

## Conclusion

The demand for energy continues to accelerate worldwide. Accordingly, the related technology is also advancing with this trend. Technological development indicates cost reduction, which also implies that energy efficiency is continuously improved. However, though traditional energy efficiency considers only the economic aspect, as the global warming is being realized and concerns about carbon emissions are deepening, the consideration of the ecological aspect is inevitable when we discuss energy efficiency.

This research investigated energy and carbon efficiency by estimating panel SFA for 21 emerging countries. To the best of our knowledge, this study is the first attempt to measure efficiency in economic and ecological aspects, particularly for emerging countries.

For the empirical model, we chose variables to explain the economic output and ecologically unfavorable output of each efficiency model. The empirical model consisted of energy consumption, gross fixed capital formation, labor force, and economic complexity index, to represent the factors of production and economic structure, respectively. We conducted SFA with the TFE model, which takes account of each country’s heterogeneity. Most of the estimated coefficients are statistically significant only except the energy consumption coefficient in the carbon inefficiency model.

In the case of the energy efficiency model, it is revealed that capital formation and labor force positively affect economic growth. On the other hand, capital variable has no impact on carbon emissions. However, energy consumption, and labor force and economic complexity index have a positive and negative effect on carbon emissions, respectively.

The efficiency score results provide some useful implications. Mexico is a representative example. While Turkey’s energy efficiency score is in third place among the 21 emerging countries, it is also the most inefficient country in the view of ecological side. If we do not consider the ecological aspect but only the economic aspect, Mexico may be classified as an energy frontier country. However, Mexico cannot be called as the real frontier country in this analysis. Egypt records the lowest score in carbon inefficiency but has a high rank of energy efficiency. Brazil is also in similar situation. Consequently, this type of countries can be qualified as frontier country.

Among top six countries in energy efficiency, Brazil, Egypt, Mexico, Czech Republic, Pakistan, Korea have almost highest carbon inefficiency score. It can be said that energy efficiency is the contrary characteristics with carbon efficiency within the analysis period given that most of energy efficient countries have relatively lower carbon efficiency score, though Brazil and Egypt are good in both energy and carbon efficiency. this is reasonable since the Levelized Cost of Electricity (LCOE) of renewable energy source has generally been higher than traditional, high carbon intensity energy source. In our analysis framework, the higher generation costs, the lower energy efficiency by decreasing the economic output.

When interpreting the results of SFA, we should be aware of the difference or growth along with time considering the methodology adopted in this research is a kind of time series analysis (exactly, a panel). For the energy efficiency and carbon inefficiency growth, the 21 emerging countries are divided into four groups (the combination of energy and carbon efficiency increase or decrease. The policy implications must be made differently according to this grouping. Only Brazil, Greece, Korea, Malaysia, Pakistan, and Thailand can be classified as the frontier country since they increase energy and carbon efficiency simultaneously.

Above these, economic output and ecological output such as carbon emissions are not only affected by the technological factor, but also economic structure. We mentioned in the introduction section, economic structure is the key factor when we estimate energy efficiency precisely since most simple standard, energy intensity cannot consider economic structure. Thus, economic structure roadmap sharing from developed to developing countries should be encouraged. As shown in the empirical results, the more complex the economy is, the more economic output and the less carbon emissions is generated. The most important thing is that global cooperation will be needed in advancing energy and carbon efficiency. International organization must be the connection between the countries to share the economic structure, energy mix, and technological research results.

Furthermore, this research propose that the policy makers must be aware of the difference between estimated energy efficiency and energy intensity as shown in Table 6. Most of countries show different ranking except a few countries such as Brazil, Greece, and Turkey. These results show that to judge the energy efficiency by using energy intensity can be dangerous. Numerous studies support this. The policy makers should consider other factors such as economic complexity index or something can represent the economic structure. In our recommendation, structure effect decomposed from energy intensity using decomposition analysis can be useful variable to estimate energy efficiency^40^.

Our empirical model takes energy usage pattern and economic structure into account by adopting fossil fuel usage and economic complexity index variable. Thus, our efficiency estimation results are different from those of energy intensity. This suggests that it is not appropriate to investigate energy efficiency with energy intensity.

To improve energy and carbon efficiency simultaneously, a country must use not only cheap but low-carbon energy sources, which is represented by nuclear energy. However, given that nuclear plants have been suppressed since the Fukushima case, energy efficiency improvement of frontier countries may not be from the extension of nuclear power plant usage. In this analysis framework, the energy and carbon efficiency scores will be reflected in the frontier if a country uses nuclear energy as the primary energy source due to its economic and low-carbon characteristics. This indicates the model’s limitations. Undoubtedly, nuclear energy sources are cost-efficient, such as uranium. however, the ecological problem except carbon emissions and the social cost of risk must be included. Furthermore, we can estimate efficiency more precisely if lower risk from well-established energy mix portfolios can be properly reflected.
